# Quantitative and Qualitative Analysis of Transient Fetal Compartments during Prenatal Human Brain Development

**DOI:** 10.3389/fnana.2016.00011

**Published:** 2016-02-24

**Authors:** Lana Vasung, Claude Lepage, Milan Radoš, Mihovil Pletikos, Jennifer S. Goldman, Jonas Richiardi, Marina Raguž, Elda Fischi-Gómez, Sherif Karama, Petra S. Huppi, Alan C. Evans, Ivica Kostovic

**Affiliations:** ^1^Department of Developmental Neuroscience, Croatian Institute for Brain Research, School of Medicine, University of ZagrebZagreb, Croatia; ^2^Division of Development and Growth, Department of Pediatrics, University of GenevaGeneva, Switzerland; ^3^Ludmer Centre for Neuroinformatics, McGill Centre for Integrative Neuroscience, Department of Biomedical Engineering, Montreal Neurological Institute, Montreal, McGill UniversityMontreal, QC, Canada; ^4^Department of Neuroscience and Kavli Institute for Neuroscience, Yale School of MedicineNew Haven, CT, USA; ^5^Laboratory of Neurology and Imaging of Cognition, Department of Neuroscience, University of GenevaGeneva, Switzerland

**Keywords:** cerebral cortex, subplate, cortical plate, human fetal brain

## Abstract

The cerebral wall of the human fetal brain is composed of transient cellular compartments, which show characteristic spatiotemporal relationships with intensity of major neurogenic events (cell proliferation, migration, axonal growth, dendritic differentiation, synaptogenesis, cell death, and myelination). The aim of the present study was to obtain new quantitative data describing volume, surface area, and thickness of transient compartments in the human fetal cerebrum. Forty-four postmortem fetal brains aged 13–40 postconceptional weeks (PCW) were included in this study. High-resolution T1 weighted MR images were acquired on 19 fetal brain hemispheres. MR images were processed using in-house software (MNI-ACE toolbox). Delineation of fetal compartments was performed semi-automatically by co-registration of MRI with histological sections of the same brains, or with the age-matched brains from Zagreb Neuroembryological Collection. Growth trajectories of transient fetal compartments were reconstructed. The composition of telencephalic wall was quantitatively assessed. Between 13 and 25 PCW, when the intensity of neuronal proliferation decreases drastically, the relative volume of proliferative (ventricular and subventricular) compartments showed pronounced decline. In contrast, synapse- and extracellular matrix-rich subplate compartment continued to grow during the first two trimesters, occupying up to 45% of telencephalon and reaching its maximum volume and thickness around 30 PCW. This developmental maximum coincides with a period of intensive growth of long cortico-cortical fibers, which enter and wait in subplate before approaching the cortical plate. Although we did not find significant age related changes in mean thickness of the cortical plate, the volume, gyrification index, and surface area of the cortical plate continued to exponentially grow during the last phases of prenatal development. This cortical expansion coincides developmentally with the transformation of embryonic cortical columns, dendritic differentiation, and ingrowth of axons. These results provide a quantitative description of transient human fetal brain compartments observable with MRI. Moreover, they will improve understanding of structural-functional relationships during brain development, will enable correlation between *in vitro*/*in vivo* imaging and fine structural histological studies, and will serve as a reference for study of perinatal brain injuries.

## Introduction

In the developing human brain, the genesis of cerebral cortex takes place in transient fetal compartments (His, 1904; O'Leary and Borngasser, 2006; Rakic, 2006; Kostović and Judaš, 2007, 2015; Bystron et al., 2008). It occurs through precise spatiotemporal gene expression of cell proliferation, cell migration, morphogenesis, dendritic differentiation, synaptogenesis, apoptosis, and myelination (Kang et al., 2011; Pletikos et al., 2014). Although corticogenic events take place in more than one fetal compartment, it was shown that some compartments have a predominant role as sites for particular neurogenetic events. On the boundary of the fetal cerebral ventricles, cells proliferate within the ventricular and subventricular zones, producing neurons and glia through mitotic divisions of cortical progenitors (for review see Bystron et al., 2008). Adjacent to the subventricular zone, the migration of postmitotic cells and the growth of axons occur in the intermediate zone (His, 1904; Rakic, 1972; Kostovic and Rakic, 1990; Bystron et al., 2008). Moving outwards, subplate compartment and marginal zone (at the surface of the developing telencephalon) are critical sites for early synaptic interaction (Molliver et al., 1973; Kostovic and Molliver, 1974; Kostovic and Rakic, 1990). In addition, due to its rich extracellular matrix the subplate compartment is also of great importance for guidance of axons (Molliver et al., 1973; Kostovic and Molliver, 1974; Kostovic and Rakic, 1990). Finally, the cortical plate, situated between subplate compartment and marginal zone, is the main locus of post-migratory cortical neuron differentiation (Mrzljak et al., 1988, 1992; Marín-Padilla, 1992). Compared to other primates, apart from its large size, the human brain during development shows a very prominent subplate compartment (Kostovic and Rakic, 1990; Judaš et al., 2013; Hoerder-Suabedissen and Molnár, 2015) and an enlarged subventricular zone (Kriegstein et al., 2006; Bystron et al., 2008; Rakic, 2009; Rakic et al., 2009). The prominent subplate and subventricular zones have been related to the greater number of neurons and connectivity combinations in humans (Kostovic and Molliver, 1974; Kostovic and Rakic, 1990; Judaš et al., 2013; Hoerder-Suabedissen and Molnár, 2015). However, the growth trajectories of these transient human fetal brain compartments have not been completely characterized.

Modern magnetic resonance imaging (MRI) methods allow excellent opportunities to follow development of transient fetal compartments *in vitro* (Kostović et al., 2002, 2014; Radoš et al., 2006; Widjaja et al., 2010) and even *in vivo* (Maas et al., 2004; Judaš et al., 2005; Prayer et al., 2006; Perkins et al., 2008; Miller and Ferriero, 2009; Rutherford, 2009; Habas et al., 2010a; Kostović et al., 2014). In MRI, T1 (longitudinal) and T2 (transverse) relaxation times rely on water protons, more specifically, on the mobility of water within the tissues. This changes dramatically during brain development. Thus, the MRI characteristics of telencephalic structures are not easily comparable between fetal and adult brain (Kostović et al., 2002; Radoš et al., 2006). Developmental histogenesis is characterized by transient changes in cellular and extracellular composition of neural tissue and is reflected as changes in MRI T1/T2 signal intensity within the specific developmental phases (Kostović et al., 2002, 2014; Radoš et al., 2006). In the developing brain, the composition and density of cells, un/myelinated axonal fiber amount, and the water percentage within extracellular matrix result in inversion of relative MRI signal intensities between “future cortex” and prospective white matter (Kostović et al., 2002; Radoš et al., 2006; Kostović et al., 2014). Yet, using MRI correlated with histology, it is still possible to define transient compartments and spatiotemporal indicators of fetal cerebral cortical development (Kostović et al., 2002; Radoš et al., 2006; Widjaja et al., 2010; Kostović et al., 2014). As seen on MRI images, from 13 PCW onwards, the cerebral wall displays five laminar compartments (Kostović et al., 2002; Radoš et al., 2006) that vary in MRI T1 signal intensity and can be easily distinguished. These are:

1. Ventricular zone (VZ) and ganglionic eminence (GE), which are composed of tightly packed proliferative cells (as seen, for example, in Nissl stained sections). VZ surrounds the entire surface of the ventricular walls and displays a spatio-temporal pattern in intensity of cell proliferation. It increases in thickness, with a peak thickness approximately around 23 PCW, and afterwards reduces to one cell thick ependymal layer (for review see Bystron et al., 2008). Due to the densely packed cell content, VZ and GE are characterized in T1 MR images with high signal intensity (Kostović et al., 2002; Radoš et al., 2006).
2. Subventricular zone (SVZ) appears 1 week before the cortical plate (for review see Bystron et al., 2008). Similar to the VZ, the SVZ is marked by densely packed dividing cells. Nevertheless, while VZ reduces in thickness during the mid-fetal period, the SVZ continues to increase in thickness (Zecevic et al., 2005). Approximately around 11 PCW, the SVZ is divided into inner and outer layers by tangentially oriented fibers (periventricular fiber rich zone) (Smart et al., 2002). Inner SVZ is characterized by high T1 MRI signal intensity. However, it is difficult to distinguish inner SVZ from VZ by MRI in all the regions of the cerebrum. Contrarily, the periventricular fiber rich zone can be seen as a low signal MRI intensity layer in some regions. The outer SVZ contains dividing neural precursors that are the principal source of cortical neurons after 25 PCW. Due to the less densely packed cells, outer SVZ cannot be discerned from the intermediate zone by MRI—outer SVZ “blends” with the intermediate zone, both showing the “moderate” T1 MRI signal intensity.
3. Intermediate zone (IZ) is a heterogeneous layer positioned between proliferative compartments and postmigratory compartments. IZ contains migrating cells and topographically organized axonal fibers. Due to the mixed content of fibers and migrating cells, IZ is characterized by moderate T1 signal intensity.
4. Subplate compartment (SP) is a transient cytoarchitectonic compartment enriched with extracellular matrix. SP is composed of subplate neurons, and “waiting” thalamocortical and cortico-cortical fibers (Kostovic and Rakic, 1990). SP is characterized by low MRI signal intensity and can be distinguished clearly on MRI images.
5. Cortical plate (CP) is composed of densely packed postmigratory cells. After the peak of neuronal migration, around 20 PCW, it displays several developmental stages that are characterized by areal, laminar and cytological differentiation. Before 30 PCW, CP is characterized by high T1 MRI signal intensity. After 28 PCW the CP starts to show VI layer divisions with ongoing dendrite differentiation resulting in decrease of T1 MRI intensity. A few months after the birth, CP starts to resemble to the adult cortex in terms of MRI T1 signal intensity (http://www.bic.mni.mcgill.ca/ServicesAtlases/NIHPD-obj2).

Despite numerous studies utilizing different imaging modalities (McKinstry et al., 2002; Maas et al., 2004; Perkins et al., 2008; Huang et al., 2009; Trivedi et al., 2009; Habas et al., 2010b; Takahashi et al., 2012; Makropoulos et al., 2014), quantitative data describing the precise growth trajectories of transient embryonic compartments is unfortunately still fragmentary (Kostović et al., 2002, 2014; Radoš et al., 2006; Huang et al., 2009, 2013; Kostovic and Vasung, 2009; Widjaja et al., 2010; Huang and Vasung, 2014). Previous MRI studies of developmental changes in total brain volume (Habas et al., 2010a,b; Kuklisova-Murgasova et al., 2011; Makropoulos et al., 2014) used coarse segmentation of gray and white matter and were not able to relate transient fetal compartments to corticogenic events. One of the reasons is that the fetal transient compartments change dynamically throughout fetal development, as previously mentioned, and show different spatiotemporal relationships with cortical histogenesis (Kostović et al., 2014).

Here we provide quantitative data on the transient fetal compartments using a normative cohort of fetal human brains. The main goal of this study was to generate new volumetric MRI parameters for the analysis of transient fetal compartments, defined on the basis of reliable histological references, as it was shown that some of these parameters are useful for the delineation of cortical growth phases and their correlation with spatiotemporal gene regulation (Kang et al., 2011; Pletikos et al., 2014). Our study had two additional specific goals: (i) to use quantitative analysis of the developmental evolution of transient developmental compartments, especially the subplate compartment, in order to better understand the role of these compartments in later stages of brain development, (ii) to test the hypothesis that the voluminous transient subplate compartment in late human fetal brain is related to extraordinary richness of growing and “waiting” fronts of cortico-cortical axons (Kostovic and Rakic, 1990). These quantitative data are necessary for studies of structural and functional consequences of intrauterine and perinatal injuries of developing human cortex (Volpe, 2009) and for understanding the selective vulnerability of these fetal neural compartments (Kostovic et al., 2014).

## Materials and methods

### Materials

Forty-four postmortem brains of human fetuses and prematurely born infants were included in the current study. Human fetuses were obtained in accordance with the Croatian federal law following the medically/legally indicated abortions or spontaneous abortions performed at the School of Medicine, University of Zagreb. Premature infants were obtained after the routine autopsy procedure. The procedure for the human autopsy material was approved and controlled by the Internal Review Board of the Ethical Committee at the School of Medicine, University of Zagreb. Consent for postmortem examination was obtained from each parent.

Only the brains of fetuses (<28 PCW) without any sign of pathology (as reported by routine pathology examination) and without known genetic abnormalities were included in the study. The brains of prematurely born infants (>28 PCW) were included if the cause of death was attributed to the sudden infant death syndrome or a respiratory disease.

The age of the fetuses and prematurely born infants was estimated on the basis of their crown–rump lengths (CRL; O'Rahilly and Müller, 1984), greatest length (caliper length without inclusion of the flexed lower limbs), and pregnancy records. In order to provide accurate age estimation of fetuses and of prematurely born infants, their age was expressed as weeks from conception (PCW), (Olivier and Pineau, 1962). After examination and age estimation the skull was removed in order to prepare the postmortem brains for MRI scanning. We invested major effort, in collaboration with the pathology department, to remove the skull without or with minimal damage to the brain tissue. Nevertheless, we could not avoid small damages of brain tissue (in three cases) or brain shape distortions.

**Figure 1:**
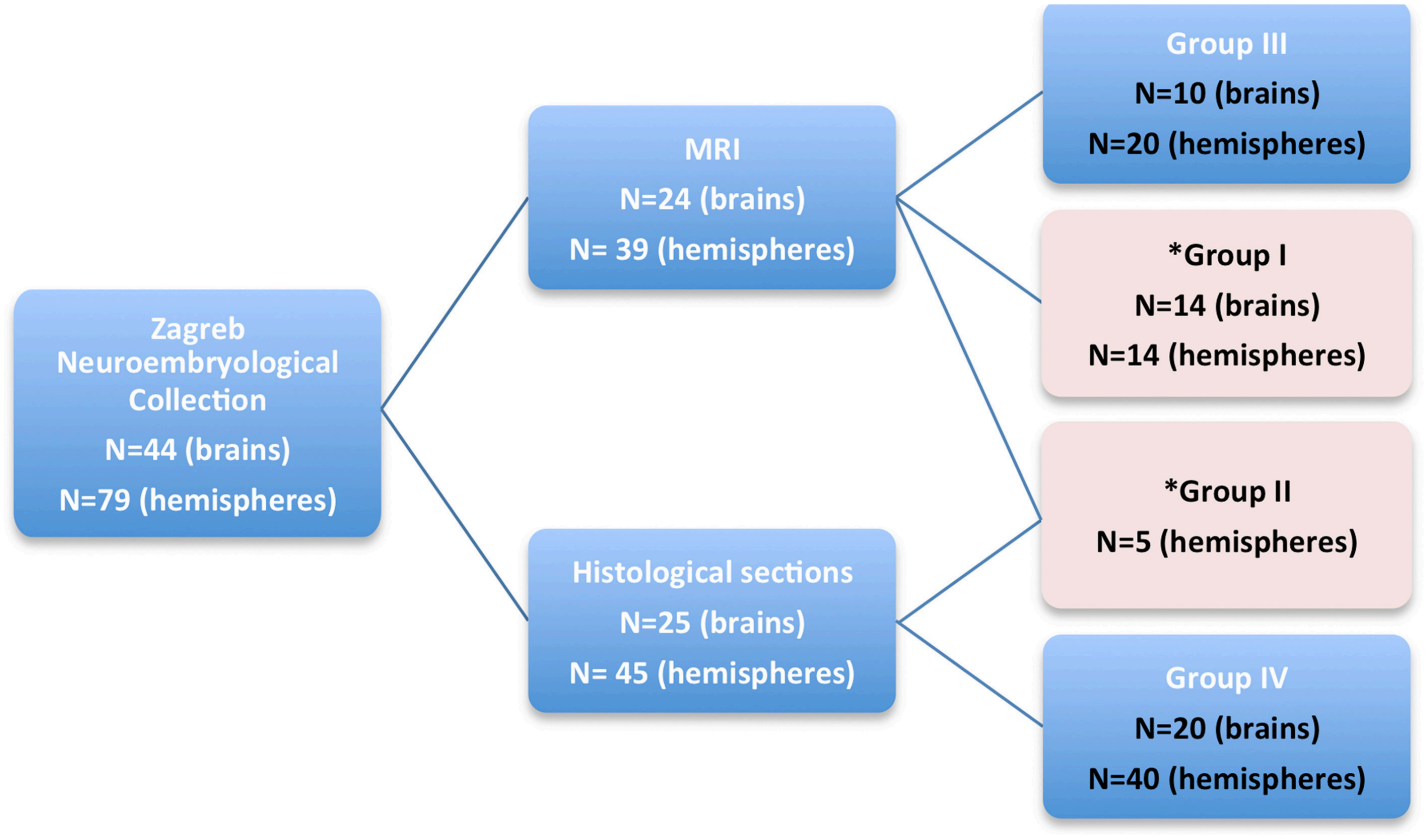
**A diagram showing four groups of subjects, taken from Zagreb Neuroembryological Collection, that were included in our study**. Quantitative MRI measurements were obtained on fetal brains marked with pink rectangle (Group I and II).

In order to broaden the quantitative and qualitative MRI analysis, and to provide a histology-based atlas, postmortem brains were divided into four groups (Figure 1):

- Group I (Quantitative MRI analysis):
  Nineteen human brain hemispheres from 14 postmortem brains (aged 13–40 PCW) were used for high-resolution quantitative MRI analysis (Table 1).
- Group II (Quantitative MRI-histology analysis):
  From Group I, we have selected five brain hemispheres (aged 13, 16, 24, 26, and 40 PCW) for histological processing. The inclusion criteria were; I. time of fixation, II. developmental phase, and III. absence of tissue damage.
- Group III (Qualitative MRI analysis):
  Ten brains (aged 11, 16, 20, 20, 21, 22, 25, 32, 37, 40 PCW) were selected and scanned with MRI in order to provide additional T1-weighted MRI properties of transient fetal compartments for quality check. T1-weighted MRI images were acquired in order to build neuroanatomical coordinate guidelines for delineation of brain structures and in order to confirm spatio-temporal MRI properties of postmortem brain during different developmental stages.
- Group IV (histology analysis):
  As an anatomical reference, needed for specimens that were not histologically processed, we have used 20 histologically processed brains [Nissl, PAS, and AChE stained sections of the fetal brains aged 13–40 PCW that are part of the Zagreb Neuro-embryological Collection (Kostovic et al., 1991; Judaš et al., 2011)]. Specimens were selected in order to serve as age-matched controls for delineation of MRI neuroanatomical structures.

**Table 1:** **Characteristics of postmortem human brains included in the quantitative MRI analysis (Group I and Group II)**.

**Age in PCW**	**Hemisphere**	**Developmental phase**	**Cause of death**
*13*	*Right*	*Early fetal phase*	*Abortus spontaneus*
16^†^	Left	Mid-fetal phase	Abortus spontaneus
*16*^†^	*Right*	*Mid-fetal phase*	*Abortus spontaneus*
18^†^	Right	Mid-fetal phase	Abortus spontaneus
18^†^	Left	Mid-fetal phase	Abortus spontaneus
19^†^	Right	Mid-fetal phase	Partus praetemporarius
19^†^	Left	Mid-fetal phase	Partus praetemporarius
20	Right	Mid-fetal phase	Abortus in tractu completus
21	Left	Mid-fetal phase	Abortus in tractu completus
21	Left	Mid-fetal phase	Abruptio placentae
*24*^†^	*Right*	*Late fetal phase*	*Abruptio placentae*
24^†^	Left	Late fetal phase	Abruptio placentae
25^†^	Left	Late fetal phase	Partus praetemporarius
25^†^	Right	Late fetal phase	Partus praetemporarius
*26*	*Right*	*Late fetal phase*	*Partus praetemporarius*
29	Right	Late fetal phase	Stillborn
30	Left	Late fetal phase	Prematurus
40	Right	Perinatal phase	Asphyxio liquris amnii
*40*	*Right*	*Perinatal phase*	*Abruptio placentae*

*The hemispheres of the same brains are marked with^†^. The hemispheres that were histologically processed (Group II) are marked in italic*.

### Methods

#### MRI acquisition

*Ex vivo* brains or separate hemispheres, with postmortem time ranging from few hours to 10 h maximally, were fixed by immersion in 4% paraformaldehyde in 0.1 M phosphate buffer, pH 7.4, and were used to obtain MR images by using the high-field 3.0T MRI device (Siemens Trio Tim). The fixation period ranged from a few weeks to a few years. As we wanted to have a uniform set of MRI signal intensity data that could be comparable between brains, one of our major concerns was the alteration of the MRI signal intensity of the brain that occurs due to the tissue fixation *per se*. In addition, formalin fixation is one of the factors that change the microstructure of the tissue, affecting and reducing the difference between the gray and white matter water. As those differences are the key to tissue discriminability using MRI, the standard three-dimensional spoiled gradient-echo (3-D GRE) sequence (magnetization-prepared rapid acquisition gradient echo -MPRAGE) failed to adequately discriminate the transient fetal compartments needed for 3D quantitative and qualitative analyses. In order to acquire the high spatial resolution and high-contrast T1-weighted postmortem fetal brain MR images, suitable for quantitative 3D MRI analysis, we had to modify commercially available VIBE sequence (volumetric interpolated brain examination (Rofsky et al., 1999). Having in mind the known challenges of postmortem MRI scanning, we have adjusted the MRI acquisition timing parameters taking into account the differences between behavior and microenvironment of water protons in the living and the formalin-fixed developing brains. Thus, we have reduced the FOV, increased the resolution and number of excitations, and finally modified the TE and TR as well as the flip angle. Finally, the parameters used for MRI acquisition were following: repetition time (TR) 14.5 ms, echo time (TE) 5.4 ms, number of excitations (NEX) 5, flip angle of 12°, acquisition time ~1.5 h per brain and section thickness ranging from 0.3 to 0.5 mm depending on the age. All brains were scanned using the wrist small-flexi eight-channel surface coil. The matrix size and the field of view were adjusted in order to obtain an isotropic spatial resolution of at least 0.3 × 0.3 × 0.3 mm^3^ for 13 PCW old fetal brains, and 0.5 × 0.5 × 0.5 mm^3^ for the fetal brains older than 15 PCW.

Variability in fixation time can result in differences in MRI signal intensities between brains, however, signal intensity differences within brains were sufficient to distinguish and delineate telecephalic structural changes resulting from microstructural events (Kostović et al., 2002; Widjaja et al., 2010). In addition, according to Tovi and Ericsson, changes in T1 due to fixation occur rapidly but stabilize after 3–4 weeks (Tovi and Ericsson, 1992), which was the minimum fixation period used in our study to ensure tissue stability and comparability between samples.

#### Histology

**Figure 2:**
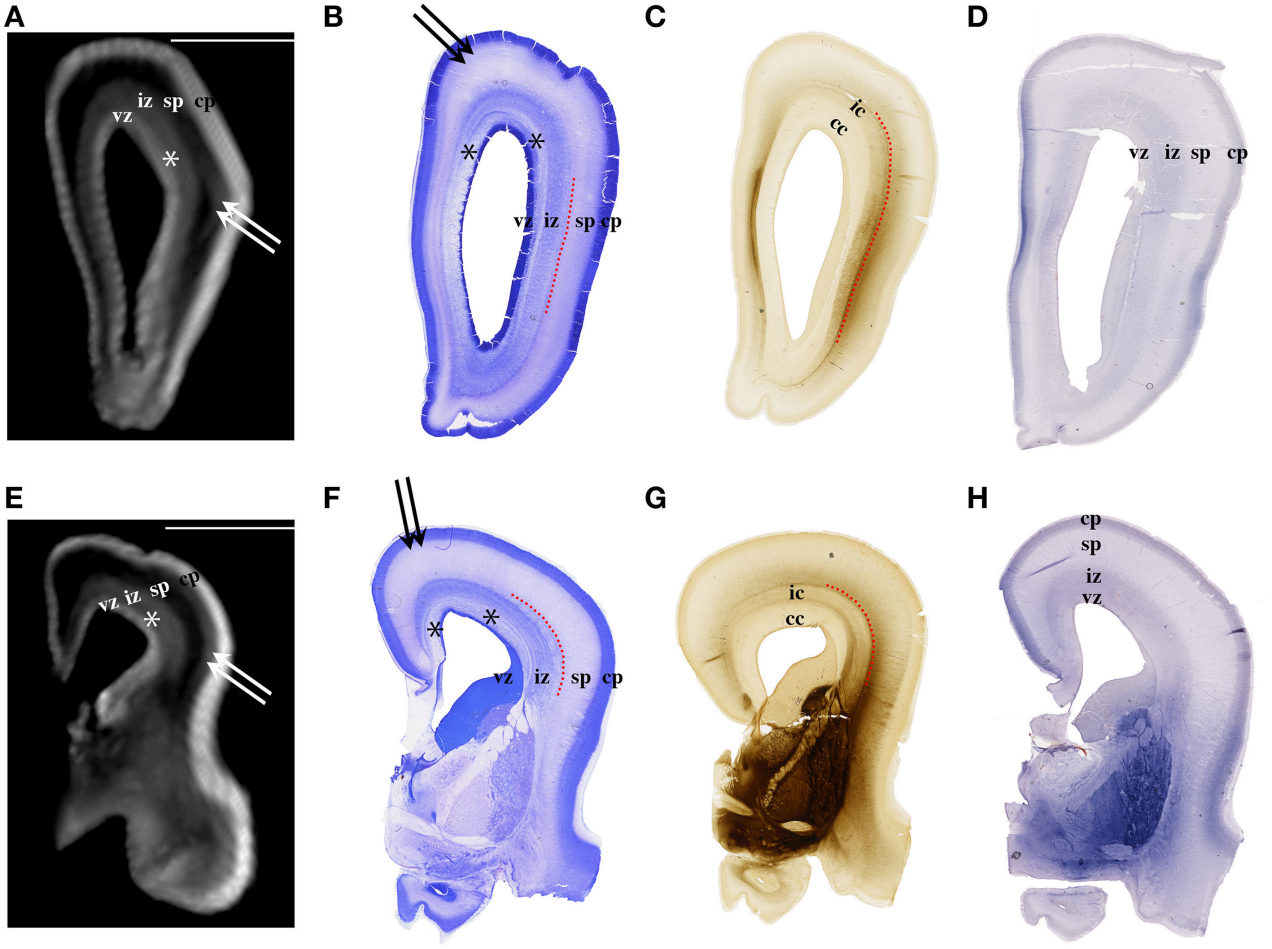
**Nissl (B,F), AChE (C,G), and CS-56 immunocytochemistry (D,H) stained coronal brain sections of the 16 PCW human fetus**. Corresponding T1-weighted MRI coronal sections of the same brain are shown in **(A,E)**. vz, ventricular zone, ^*^periventricular fiber rich layer of subventricular zone; iz, intermediate zone; sp, subplate compartment; cp, cortical plate. External capsule and its radiations are marked by a red dotted curved line **(B,F,C,G)**. Double arrows in **(A,B,E,F)** indicate the upper subplate compartment. Curved dashed red line in **(B,C,F,G)** illustrates where the border between subplate and intermediate zone was placed during manual segmentation. Scale bar = 10 mm **(A,E)**.

**Figure 3:**
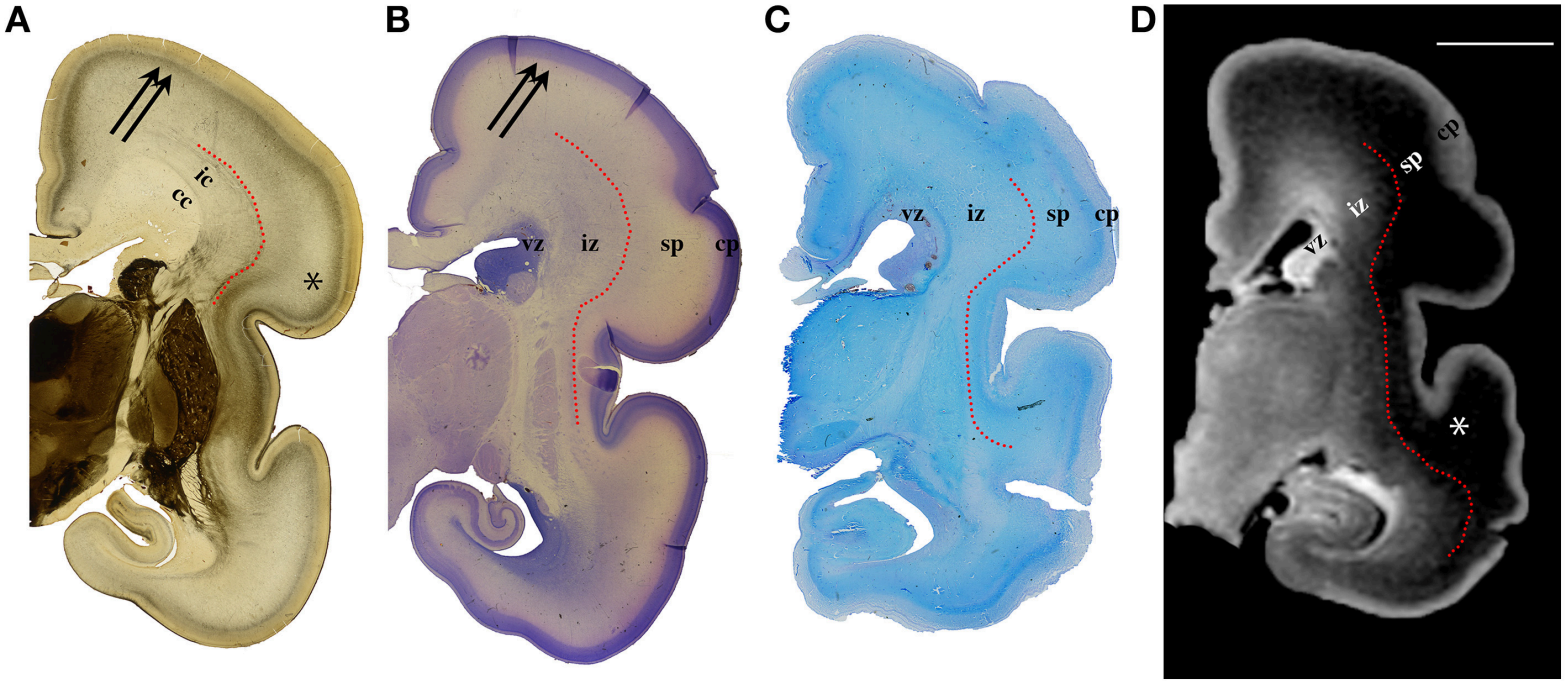
**AChE (A), Nissl (B), PAS (C) stained coronal brain sections with a corresponding T1-weighted MRI slice (D), of the human 24 PCW old fetuses**. ic, internal capsule; cc, corpus callosum; vz, ventricular zone; ^*^subplate compartment; iz, intermediate zone; sp, subplate compartment; cp, cortical plate. External capsule and its radiations are marked by a red dotted curved line **(A)**. Double arrows in **(A,B)** indicate the upper subplate compartment. Curved red line in **(A–D)** illustrates where the border between subplate and intermediate zone was placed during manual segmentation. Scale bar = 10 mm **(D)**.

Five histologically processed brains, aged 13, 16, 24, 26, and 40 PCW (Table 1) were selected as major representatives for specific phases of prenatal brain development (Kostovic and Vasung, 2009). After the MRI acquisition these brains were embedded in paraffin and serially sectioned with coronal slice thickness of 15–20 μm. Cresyl violet and Periodic Acid Schiff–Alcian Blue (PAS-AB) were used for tissue staining. The PAS histochemical staining was conducted for the visualization of acid-sulphated glycoconjugates (Vacca et al., 1978) which provided us a “gold” standard for visualization of subplate compartment, known to have an abundant extracellular matrix (ECM). In addition, neighboring Nissl-stained celloidin sections were used as guidance for delineation of the cytoarchitectonic boundaries and cellular compartments of the human fetal telencephalon. AChE stained sections (acetyl-cholinesterase histochemistry) were used in the brains that arrived to our Institute within 24 h after death. This was done with a goal to show growing thalamocortical afferents and external capsule (Kostovic and Goldman-Rakic, 1983), which have been recognized as a relatively constant border between subplate and intermediate zone (Kostović et al., 2002). Images of histological sections were captured using a charge-coupled device (CCD) camera or Nikon scanner (Figures 2, 3) and were processed using Adobe Photoshop®.

#### Image pre-processing

We have adapted and calibrated MR imaging tools, initially developed at the MNI (Montreal Neurological Institute) for processing of adult brains, and we have developed a semi-automated pipeline for processing postmortem fetal brain MR images. First, the images were manually cropped to minimize the field of view. The images were afterwards resampled at isotropic voxel sizes of 0.15 mm (age ≤ 13 PCW) or 0.25 mm (age ≥15 PCW). MRI signal intensity nonuniformity, resulting from field non-homogeneities, were corrected using the N3 method (Sled et al., 1998) with a small spline distance of 5 mm. A tissue mask was obtained by thresholding above background.

##### Tissue classification based on qualitative MRI and histology

Due to the specific anatomical organization of the human fetal brain, modifications to available processing tools designed for adult and postnatal brains are required to extend existing MRI analysis to prenatal brain. One of the major reasons for modifications, as mentioned above, are the age-dependent changes in T1 MRI signal intensity of transient fetal laminar compartments and inversion of relative signal intensities between “cortex” (high T1 signal intensity) and prospective white matter (moderate T1 signal intensity). In order to process the fetal and premature infant postmortem brains, we have developed a pipeline combining existing automatic MNI tools with several steps requiring semi-automatic and/or manual editing.

An initial tissue classification is performed using the artificial neural network (ANN) algorithm with manually selected tag points for each of the tissue classes (I = background, II = formalin, III = “cortical plate,” IV = “prospective white matter with basal ganglia”). At least 100 points per tissue class are taken for a reliable estimation of its mean intensity and variance (Zijdenbos et al., 1998; Tohka et al., 2004). In comparison to an adult brain, in the fetal brains the partial volumes of CP-Formalin in deep sulci, of white matter-like intermediate intensities, were often mis-classified as white matter. This was caused by the narrowness of the sulci and the inversion of T1 intensities (CP- high T1 signal intensity, formalin and SP-low T1 signal intensity). Consequently, the segmented images needed to be manually corrected to account for partial volume effects for formalin in narrow sulci, for correction of artifacts (tissue damage), and for masking out unwanted tissues that were attributed to the background (brain stem, pons, mesencephalon, and cerebellum). Using the Display module (MNI toolkit), semi-automatically classified tissues were manually corrected and the narrow sulci were painted in order to extract the cortical plate surface.

After semi-automatic classification, tissues labeled as class IV (prospective white matter with basal ganglia) were extracted and manually painted introducing the five new tissue classes, namely: IV = subplate, V = intermedial zone, VI = proliferative compartments, VII = subcortical gray matter, and VIII = diencephalon. The example of tissue classification on coronal slices can be seen in Figures S1–S3. Although visible, SVZ does not show a continuous 3D appearance on MRI. Therefore, the SVZ was partially classified as VZ (inner subventricular zone), and as an IZ (outer subventricular zone that could be easily traced after the appearance of T1 hypointense periventricular fiber rich zone). After 35 PCW, we could not continuously distinguish the SP from IZ, although we have observed regional differences in MRI signal intensity. Thus, after 35 PCW, SP and IZ were measured together and were classified as one compartment (IV + V) called “fetal white matter.”

Volumes of the semi-automatically segmented fetal compartments were calculated by multiplying the number of voxels with the voxel unitary volume. In order to account for the effect of minimal tissue shrinkage caused by formalin fixation [(shrinkage of 2.7–3.5% as reported by Boonstra et al., 1983; Schned et al., 1996)], we have calculated absolute but also relative volume ratios for tissue classes III–VIII. Relative volumes were expressed as a percentage of the total telencephalic volume of the hemisphere (not including the diencephalon) or as a percentage of the cerebral volume of the hemisphere (including the diencephalon).

##### Surface extraction and registration

Manual pre-alignment of fetal hemispheres in MRI scans to stereotaxic space was a prerequisite for registration of surfaces and outer surface extraction. Each brain hemisphere was first manually registered to a stereotaxic space defined on an adult human template (ICBM152). Registration of the brain hemisphere to ICBM152 stereotaxic space was performed in Register, a GUI module developed at the MNI. For registration of fetal scans with adult templates, we manually defined 10 anatomical tag points on fetal brain scans with their corresponding counterparts on the ICBM152 model. Each brain hemisphere was then co-registered to the adult model using three translations, three rotations, and one scaling option.

The extraction of inner surfaces from CP and SP, and the CP-pial boundary was fully automated (MacDonald et al., 2000; Kim et al., 2005) and was based on the previously described segmented images. Surfaces were extracted by hemisphere, with 81920 triangles and 40962 vertices. The gyrification index (GI, the ratio of total to exposed area of the pial surface) was evaluated at the pial surface of the cortical plate.

Given the rapid growth and change in fetal brain morphology between 13 and 40 weeks of gestation, it was not possible to define a global static surface model to use for surface registration, as can be done with adult brain. Fetal surfaces were instead longitudinally registered by age with reference one to another. The oldest subjects, near 40 PCW, were registered to the reference adult template (ICBM152), thus defining the latest standard stereotaxic space (Robbins, 2004; Lyttelton et al., 2007; Boucher et al., 2009). On the younger brains, the longitudinal surface registration was driven by matching borders of lobes (or regions) as manually delineated on the extracted “inner” surfaces.

**Figure 4:**
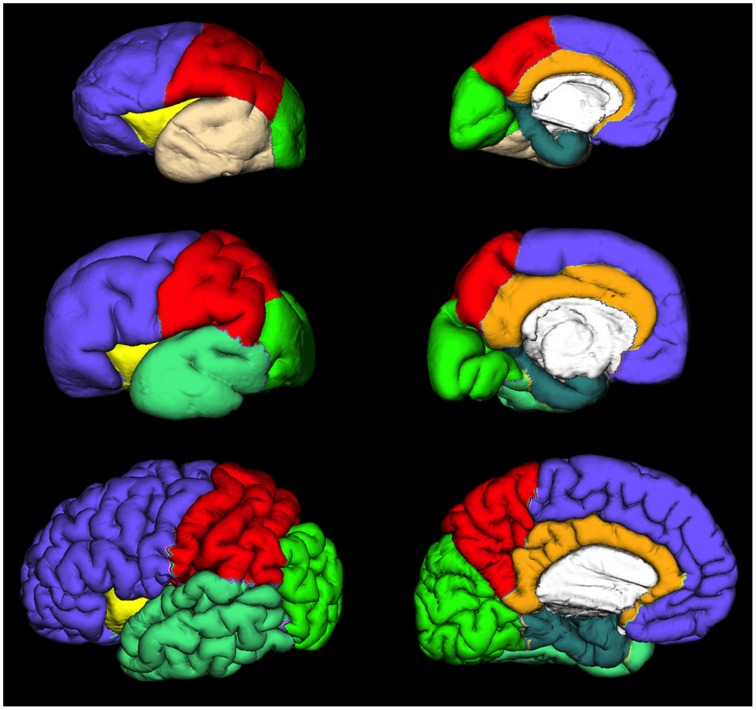
**Extracted lateral and medial cortical plate surfaces in 25 (upper row), 30 (middle row), and 40 (bottom row) PCW old fetal brains**. Regional and lobar segmentation was performed manually and the surfaces were divided into 6 lobes: frontal lobe (violet), parietal lobe (red), occipital lobe (green), temporal lobe (beige or jungle green), outer ring of the limbic lobe [gyrus fornicatus encompassing: parahippocampal gyrus (celadon green) and cingulate gyrus with subcallosal area (orange)], and insular lobe (yellow).

Regional and lobar segmentation was performed manually on the inner surfaces of the CP. The surfaces were first divided into 6 lobes [frontal lobe, parietal lobe, occipital lobe, temporal lobe, outer ring of the limbic lobe (gyrus fornicatus), and insular lobe]. The outer ring of the limbic lobe was further split into parahippocampal gyrus and cingulate gyrus resulting with 7 segmented regions in total (Figure 4). After longitudinal alignment of the surfaces, pathlines can be traced in time along the registered vertices of the individual surfaces to observe growth patterns of CP and SP both globally and regionally. For the segmentation of SP and CP, we have used anatomical borders on the inner cortical plate surfaces, i.e. gyri and sulci, which have been used for adult cortical surface segmentation, as described by von Economo and Brodmann (Brodmann, 1909; von Economo and Koskinas, 1925). The CP and SP were segmented only after identifying the primary sulci in fetal brain (Kostovic and Vasung, 2009). The anatomical borders used for surface segmentation were as follows (Figure 4).

##### Frontal lobe

The Sylvian fissure (SF) forms early in fetal development (9–13 PCW). The circular sulcus of insulae (CSI) forms during early fetal and mid-fetal development. During late mid-fetal development, the central sulcus (CS) begins to appear and can be identified continuously in the rostro-caudal direction (Kostovic and Vasung, 2009). Thus, CS, SF, and CSI provide anatomical borders of frontal lobe at lateral aspect. On the medial aspect, the frontal lobe extends to the cingulate sulcus that is continuous in appearance already in the mid fetal phase. The parolfactory sulcus does not appear before the early preterm phase, so from the rostral end of the cingulate sulcus we have extrapolated the line that most resembles the adult parolfactory sulcus (connecting rostral end of the cingulate sulcus with substantia perforata anterior) dividing the subcallosal area from frontal lobe.

##### Temporal lobe

As there is no sulcus delimiting the temporal from occipital lobe in fetal brain, we have extrapolated line connecting the Sylvian fissure with occipitotemporal incisures on the lateral aspect. On the medial aspect, the temporal lobe was delimited by collateral sulcus.

##### Occipital lobe

The borders of the occipital lobe were defined as follows: on the lateral aspect the extrapolated lines connecting the occipito-temporal incisures and parieto-occipital fissure with the Sylvian fissure, on the medial aspect occipitotemporal incisures and parieto-occipital fissure.

##### Parietal lobe

On the lateral aspect, the parietal lobe is delimited by the central sulcus and extrapolated line connecting the parieto-occipital fissure with the Sylvian fissure. On the medial aspect, the parietal lobe is delimited by the parieto-occipital fissure and cingulate sulcus.

##### Insular lobe

The circular sulcus of the insula provided a clear border between the insula and the frontal, parietal, and temporal lobes.

##### Outer ring of the limbic lobe (gyrus fornicatus) encompassing

- Gyrus cynguli and area subcallosa
  Delimited by cingulate sulcus dorsally and callosal sulcus ventrally.
- Parahippocampal gyrus, uncus, and substantia perforata anterior
  The collateral sulcus and rhinal sulcus were defined as borders between gyrus parahippocampalis and the remaining temporal lobe. The border between isthmus of gyrus cinguli and gyrus parahippocampalis was an extrapolated line connecting the most inferior part of splenium of the corpus callosum with the parieto-occipital fissure.

##### Telencephalic measurements

The thickness of each CP and SP was defined at the vertices of each surface.

Thickness was measured by taking the absolute distance between corresponding vertices on each surface. It was blurred, on the surface in its native space, with a 5 mm kernel for brains < 20 PCW and 10 mm kernel for brains >20 PCW (fwhm) (Boucher et al., 2009). This was done in order to increase signal-to-noise ratio. We have used smaller values for blurring than those applied in adults (20–30 mm) because of the size of the fetal brains.

Measurements of the surface area, volume, and average thickness of the CP (13–40 PCW) and SP (21–30 PCW) were taken for each segmented lobe and region. The surface areas (mm^2^) and volumes (mm^3^) of the CP and SP in different regions and lobes were calculated by first evaluating elemental areas and volumes at the vertices, then summing these measures over the vertices defining each lobe or region. For an elemental area, the area of a surface triangle is distributed equally (weight 1/3) to each of its three vertices. Similarly, an elemental volume is calculated from the volume of the prism formed by the linked vertices of each triangle pair between the two surfaces. As for volumetric measures, the lobar volumes are expressed as an absolute value or as a percentage they occupy in the specific fetal compartment (CP or SP).

Statistical analysis was performed using the software SPSS® and Matlab®. Detailed description of each analysis is provided in the results.

## Results

### Relationship between postconceptional weeks and total volume of transient fetal compartments

We have used the Spearman correlation in order to test the correlation between age and volume of transient fetal compartments. As expected we found significant positive correlations between age (in PCW) and absolute volume of the hemisphere (*r*_s_ = 0.953, *N* = 19, *p* < 0.01), cortical plate (*r*_s_ = 0.937, *N* = 19, *p* < 0.01), subplate compartment for the period from 13 to 30 PCW (*r*_*s*_ = 0.935, *N* = 17, *p* < 0.01), intermediate zone (*r*_s_ = 0.897, *N* = 19, *p* < 0.01), volume of subcortical gray matter (*r*_s_ = 0.963, *N* = 19, *p* < 0.01), and diencephalon (*r*_s_ = 0.955, *N* = 19, *p* < 0.01). The absolute volume of proliferative compartments showed significant positive correlation with developmental age from 13 to 25 PCW (*r*_*s*_ = 0.917, *N* = 12, *p* < 0.01). After 25 PCW, when the peak of volume is reached, the volume of proliferative compartments showed negative correlation with developmental age, that is, a rapid decline (*r*_s_ = −0.774, *N* = 7, *p* = 0.04).

Although it is known that these compartments increase in their volume with age, until now there were no reports on what hemispheric percentage these compartments occupy at the given postconceptional age. For that purpose we took into account the entire volume of the telencephalic hemisphere and we have expressed the volume of each compartment as a percentage of the total volume of the telencephalic hemisphere. We have used the Spearman correlation in order to test the correlation between age and relative volume of transient fetal compartments. The only significant correlations were found between age and relative volume of proliferative compartments (a negative correlation with *r*_s_ = −0.931, *N* = 19, *p* < 0.01) and relative volume of subplate compartment between 13 and 30 PCW (a positive correlation with *r*_s_ = 0.877, *N* = 17, *p* < 0.01). This suggests that the relationship between age and percentage of the hemisphere occupied by certain transient fetal compartment may not be linear.

**Figure 5:**
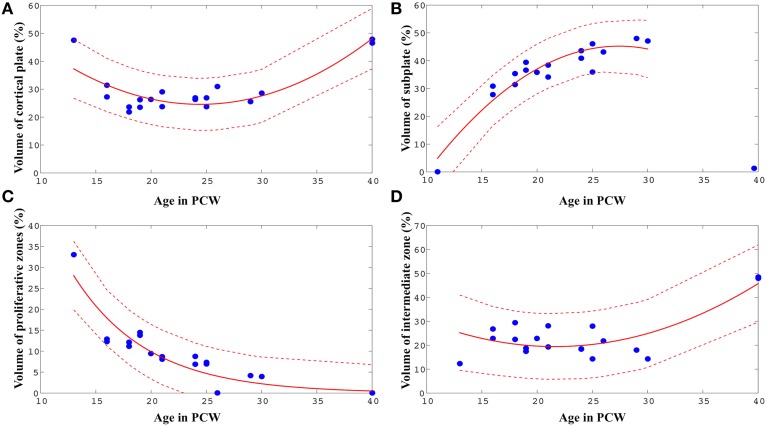
**Best-fit curves (red lines) with 95% prediction bands (between the dashed red lines) for the relations between age in PCW and relative volumes of cortical plate (A), subplate (B), proliferative compartments (C), and intermediate zone (D) within one hemisphere of the telencephalon**.

Therefore, in order to reveal the nature of a relationship between age and relative volumes of transient fetal compartments, we fit non-linear models (second-order polynomial, exponential, and Gaussian), using Matlab. For every fit, we chose between the three functional forms based on the adjusted *r*^2^ value. In all cases, these models provided better fits than a simple linear model. The best-fit parameter values are shown in (Figure 5).

The relative volume of the cortical plate was best predicted from age by using a best-fit 2nd degree polynominal curve (Figure 5A, V${}_{\text{Cortical}\_\text{plate}}=0.10^{*}$ PCW^2^ ± 4.75^*^ PCW + 82.59: adjusted *r*^2^ = 0.74). For the prediction of the relationship between the relative volume of the subplate compartment and age (only between 13 and 30 PCW), a 2nd degree polynominal curve was found to be the most adequate fit (Figure 5B, V${}_{\text{subplate}}=0.15^{*}$ PCW^2^+8.21^*^ PCW ± 67.32: adjusted *r*^2^ = 0.87). The relative volume of intermediate zone was also best predicted from age by using a best-fit 2nd degree polynominal curve (Figure 5D, V${}_{\text{Intermediate}\_\text{zone}}=0.08^{*}$ PCW^2^ ± 3.37^*^ PCW + 55.84: adjusted *r*^2^ = 0.60). An exponential model showed to be the most appropriate for the prediction of the relationship between relative volume of proliferative fetal compartments and age (Figure 5C, V${}_{\text{proliferative}}=194.78^{\left(-0.15*\text{PCW}\right)}$, adjusted *r*^2^ = 0.84). Finally, in order to assess the relationship between relative volume of diencephalon (percentage of total volume of telencephalon and diencephalon of one hemisphere) and age, we have used best-fit 2nd degree polynomial curve (V${}_{\text{Diencephalon}}=0.01^{*}$PCW^2^ ± 0.87^*^PCW + 16.01, adjusted *r*^2^ = 0.60).

### Changes in thickness of cortical plate and subplate during prenatal brain development

**Figure 6:**
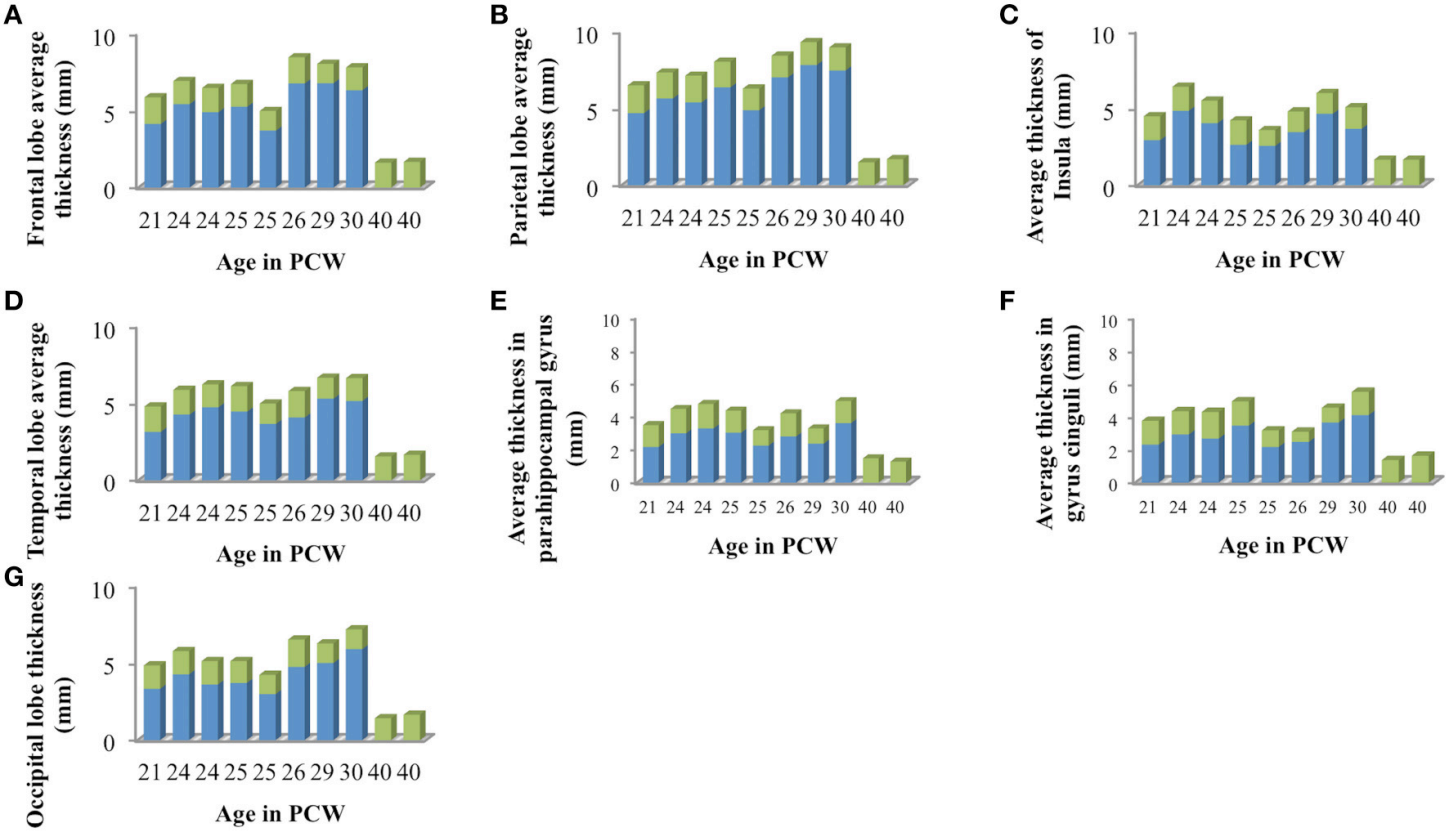
**Mean cortical thickness of cortical plate (green) and subplate compartment (blue) in frontal lobe (A), parietal lobe (B), insular lobe (C), temporal lobe (D), in the outer ring of the limbic lobe [gyrus fornicatus encompassing: parahippocampal gyrus (E) and cingulate gyrus with subcallosal area (F)], and occipital lobe (G) measured in millimeters**.

Mean thickness of CP and SP in segmented lobes and regions was measured in 10 brains (21–40 PCW, Figure 6) since we have detected the appearance of the primary sulci at this time [described also by Chi et al. (1977)].

**Figure 7:**
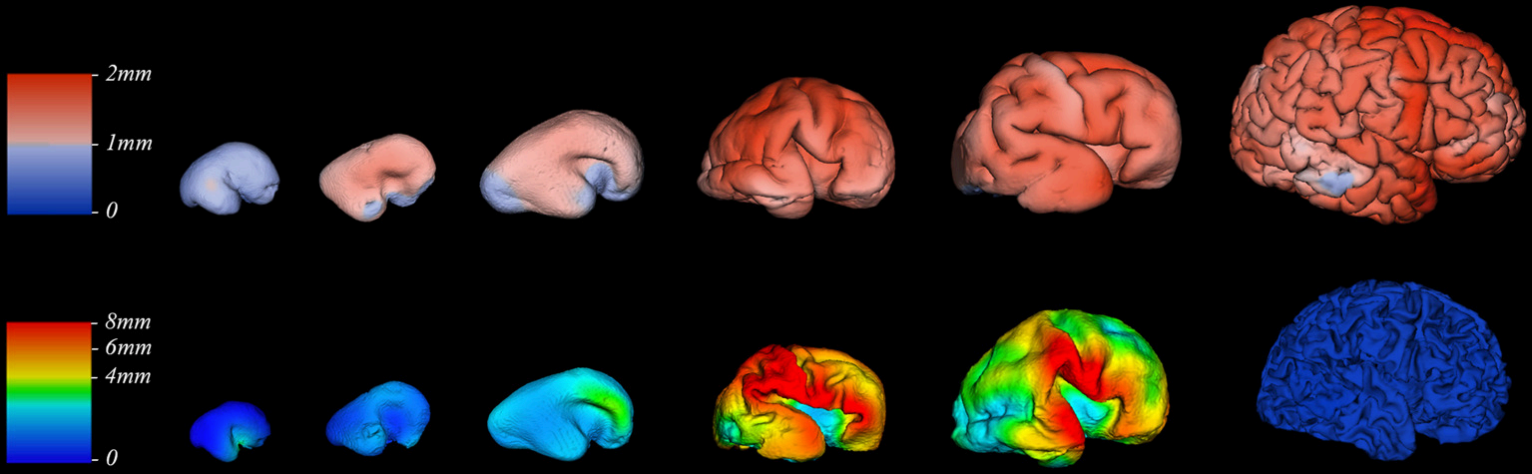
**Thickness of cortical plate (upper row) and subplate compartment (bottom row) measured in millimeters (color coded bars) at 13, 16, 18, 24, 30, and 40 PCW (left to right)**.

We did not find significant correlation between PCW and mean cortical plate thickness of seven segmented cortical plate areas (Figure 6). Moreover, curve fitting did not reveal any satisfactory model (low *r*^2^) describing age dependent changes in the mean cortical plate thickness of segmented regions. This might be due to the small sample size, below resolution sub-millimeter changes in cortical thickness during prenatal brain development, or due to the changes in cortical thickness that do not have lobar predominance (Figures 6, 7). Spatio-temporal changes of cortical plate thickness across all vertices throughout the hemisphere have been calculated in all subjects and can be seen in Figure 7 (upper row).

Since we could not detect the subplate compartment continuously at 40 PCW it was approximated to 0 mm. We have found significant positive correlation between PCW (in the period between 13 and 30 PCW) and mean subplate plate thickness of five segmented subplate areas (Figure 6; *r* = 0.884, *N* = 8, *p* = 0.004 for the parietal lobe thickness, *r* = 0.828, *N* = 8, *p* = 0.011 for the occipital lobe thickness, *r* = 0.73, *N* = 8, *p* = 0.04 for the frontal lobe thickness, *r* = 0.774, *N* = 8, *p* = 0.024 for the cingulate gyrus thickness, and *r* = 0.821, *N* = 8, *p* = 0.012 for the temporal lobe thickness). The average thickness of insula and parahippocampal gyrus did not show significant correlation with PCW. Mean subplate thickness reached maximal value at 30 PCW in all segmented areas. Spatio-temporal changes of subplate thickness across all vertices throughout the hemisphere can be seen in the Figure 7 (bottom row).

### Regional surface growth of cortical plate during prenatal development

The total surface area of cortex, from 13 to 40 PCW, showed significant and strong positive correlation with age (*r*_s_ = 0.98, *N* = 19, *p* < 0.01). In addition, the level of gyrification (Figure 7), calculated as gyrification index, was also significantly correlated with PCW (*r*_s_ = 0.5, *N* = 19, *p* = 0.03).

**Figure 8:**
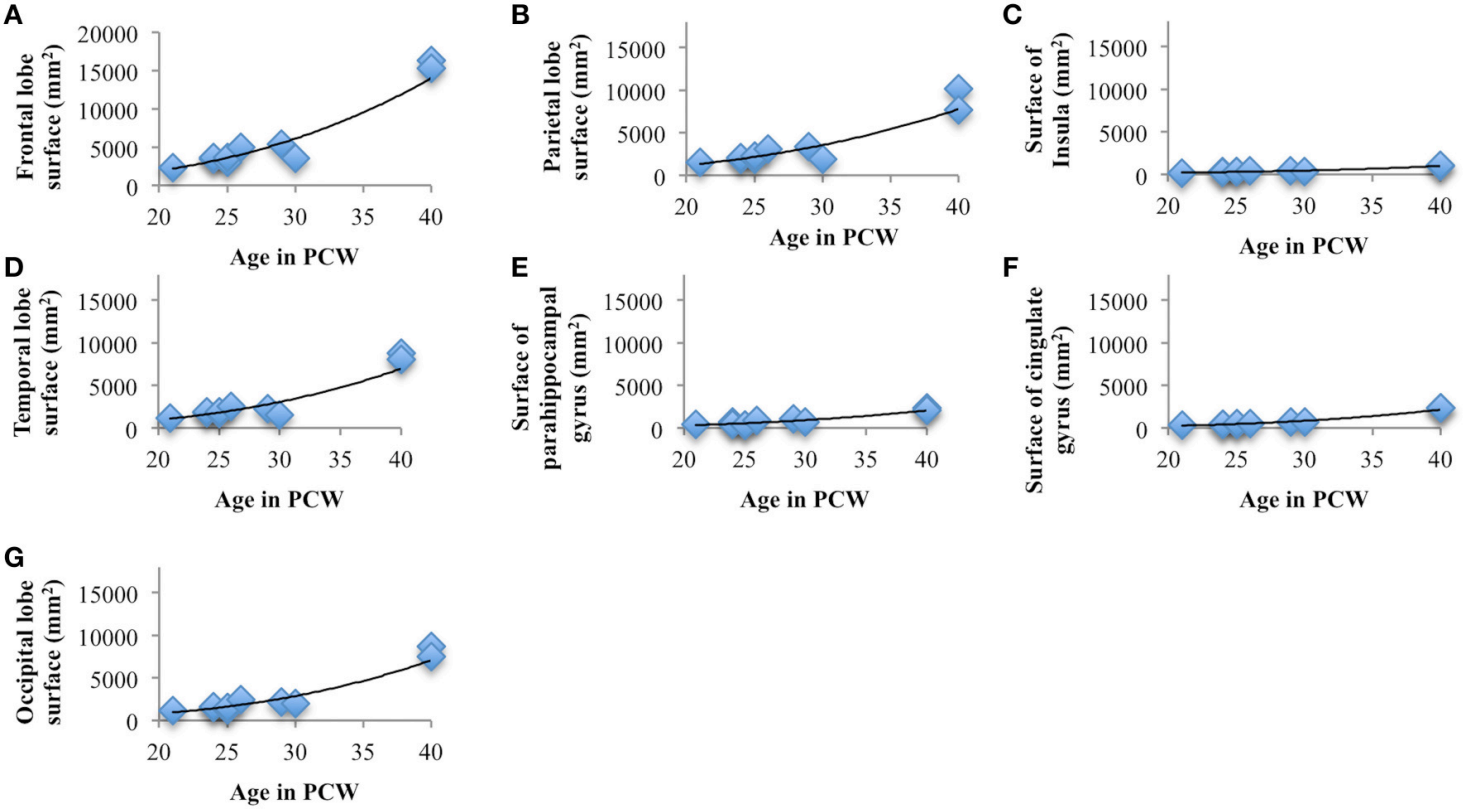
**Line charts showing the increase of cortical plate surface area of frontal lobe (A), parietal lobe (B), insular lobe (C), temporal lobe (D), outer ring of the limbic lobe [gyrus fornicatus encompassing: parahippocampal gyrus (E) and cingulate gyrus with subcallosal area (F)], and occipital lobe (G) during prenatal brain development**.

Surface areas across seven cortical plate regions during development were not normally distributed, as revealed by Shapiro-Wilk test. Spearman's rank-order correlation revealed significant positive correlation between age and surface area of parietal lobe (*r*_s_ = 0.82, *N* = 10, *p* = 0.004), occipital lobe (*r*_s_ = 0.87, *N* = 10, *p* = 0.001), frontal lobe (*r*_*s*_ = 0.77, *N* = 10, *p* = 0.009), temporal lobe (*r*_s_ = 0.67, *N* = 10, *p* = 0.03), insula (*r*_s_ = 0.91, *N* = 10, *p* < 0.01), cingulate gyrus (*r*_s_ = 0.924, *N* = 10, *p* < 0.01), and parahippocampal gyrus (*r*_s_ = 0.77, *N* = 10, *p* < 0.01; Figure 8). Nevertheless, the relative surface of six segmented areas (percentage of the total hemispheric surface of CP) did not show significant correlation with age [except the weak positive correlation between the relative surface of cingulate gyrus and age (*r*_s_ = 0.65, *N* = 10, *p* = 0.04)]. The largest portion of cortical plate surface was occupied by frontal lobe (~34%), followed by parietal and temporal lobes (~20%). The relative surface areas of six segmented regions remained unchanged during development.

### Coordinated changes of cortical plate and subplate volumes during prenatal human brain development

**Figure 9:**
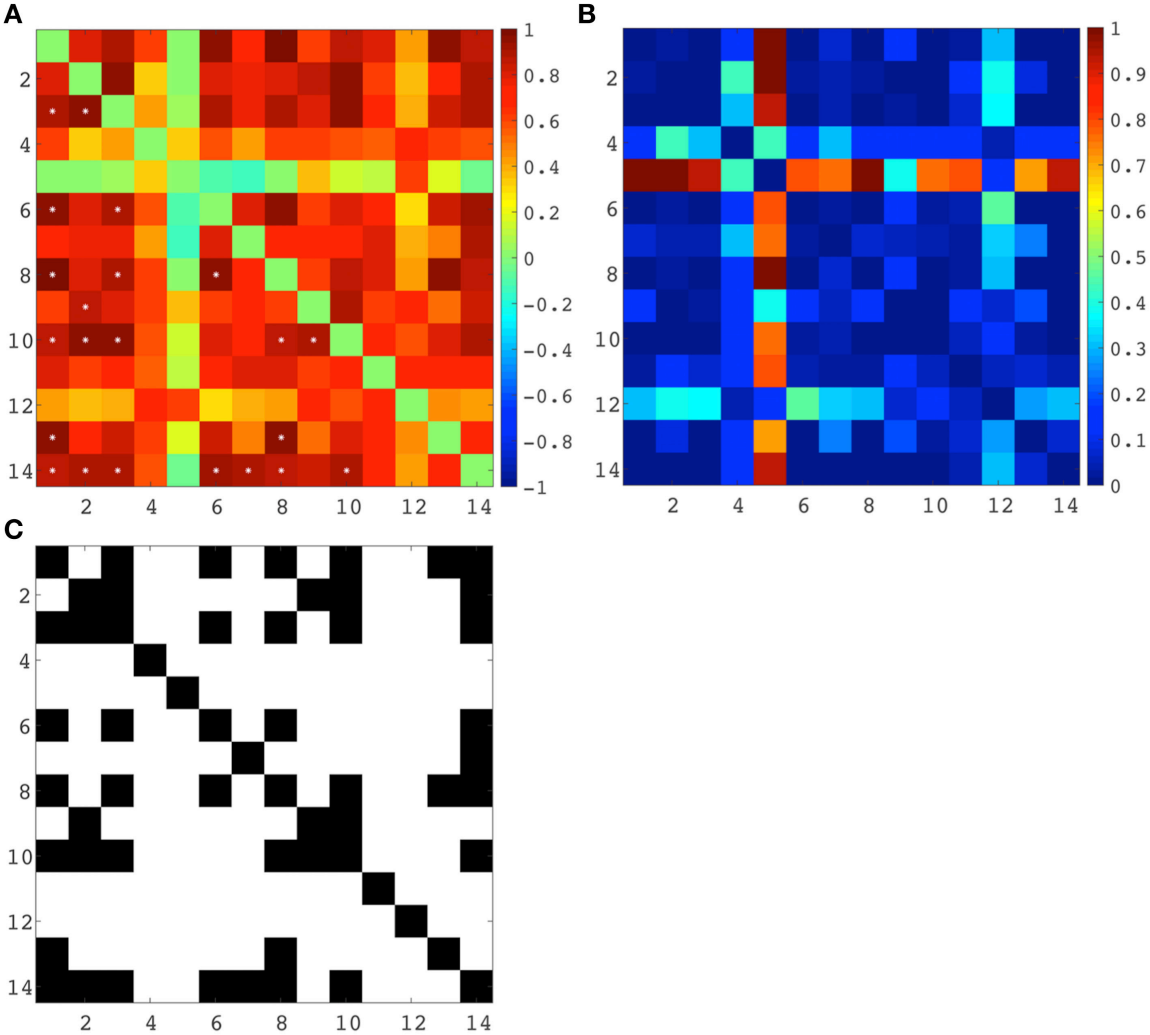
**(A) Correlation matrix (Spearman's correlation coefficient - color code on the right) between all seven regional volumes of cortical plate and subplate across eight subjects, each at a different age (21–30 PCW)**. Significant correlations are marked with ^*^. **(B)** Uncorrected *p*-values for the correlation coefficients. **(C)** Significant correlations (FDR-adjusted *p*-value < 0.05), where a black matrix entry indicates significance. Regional volumes of the cortical plate are marked with numbers 1–7 [parietal lobe (1), occipital lobe (2), frontal lobe (3), insula (4), cingulate gyrus (5), temporal lobe (6), and parahippocampal gyrus (7)]. Regional volumes of the subplate compartment are marked with numbers 8–14 [parietal lobe (8), occipital lobe (9), frontal lobe (10), insula (11), cingulate gyrus (12), temporal lobe (13), and parahippocampal gyrus (14)].

Spearman correlation coefficients were computed between all regional and lobar volumes of cortical plate and all regional and lobar volumes of subplate across eight subjects, each at a different age (from 21 to 30 PCW), yielding a 14 × 14 correlation matrix and corresponding *p*-values (Figure 9). The significance level was set at 0.05, and the *p*-values were adjusted for multiple comparisons using False Discovery Rate. This resulted in 22 significant correlations (Figure 9C). Positive correlations between cortical plate of frontal lobe and parietal lobe, between frontal lobe and occipital lobe, between frontal lobe and temporal lobe, and between temporal lobe and parietal lobe are significant across ages (Figure 9A, asterisks, Figure 9C). Furthermore, we have also found significant positive correlations across ages between the volume of subplate of frontal lobe and parietal lobe, frontal lobe and occipital lobe, frontal lobe and parahippocampal gyrus, parietal lobe and temporal lobe, and between subplate of parietal lobe and parahippocampal gyrus (Figure 9A, asterisk, Figure 9C).

As expected the volume of the subplate of frontal, occipital and parietal lobe showed significant positive correlation with the volume of the cortical plate of the same lobes (Figure 9A, asterisk, Figure 9C). Moreover, the volume of the subplate of frontal lobe showed significant positive correlation also with the cortical plate volume of parietal and occipital lobe, while the volume of the subplate of parietal lobe showed significant correlation with cortical plate of frontal and temporal lobes (Figure 9C).

## Discussion

In this study we provide quantitative data on individual transient fetal compartments, such as thickness, as well as total volume, surface area, and gyrification of human brain during development. Using MRI aligned to histological sections, we show growth trajectories of corticogenic regions during human mid-fetal and late fetal periods of cerebral development. These findings are consistent with general embryological data and previous knowledge on timing of intrauterine corticogenic events in humans (Kostovic and Rakic, 1990; Bystron et al., 2008; Kostovic and Vasung, 2009; Kostović and Judaš, 2015). We have observed significant positive correlation between developmental age and absolute volume of cortical plate, intermediate zone, subcortical gray matter, and diencephalon but also between developmental age and subplate from 13 to 30 PCW, and developmental age and proliferative compartments from 13 to 25 PCW. However, the percentage of hemispheric volume occupied by transient fetal compartments did not show correlation with age, except for relative volumes of proliferative compartments, which showed a negative relationship with age, and relative volumes of subplate compartment, which showed a positive relationship with age from 13 to 30 PCW. These results indicate the importance of these transient compartments during the reorganization of the prenatal human brain. Thus, we have obtained quantitative indicators of transient corticogenic compartments, which are useful for better neurobiological interpretation of existing and future developmental MRI data.

### Volume of transient fetal compartments as an indicator of intensity of histogenetic events

The results of this study demonstrate that precise histological delineation of transient fetal compartments based on different histological, histochemical, and cytological methods (Kostovic and Rakic, 1990; Kostović et al., 2002; Widjaja et al., 2010; Huang et al., 2013) discloses reliable anatomical landmarks for corresponding MR images (Figures 2, 3) and allows volumetric measurements of individual transient compartments. The proliferative (ventricular and subventricular) compartments decrease in size and hemispheric percentage occupied after 25 PCW (Figure 5) indicating the cessation of neurogenesis and switch to gliogenesis (Bystron et al., 2008). However, the developmental neurological interpretation of growth curves for the relative volumes of other transient compartments is not as straightforward.

Growth curves and changes in thickness of cortical plate reported here are likely difficult to interpret due to the dynamic addition of neurons to increasingly superficial positions of cortex (Rakic, 1982, 1988), regional differences in lamination of isocortical and alocortical (limbic) regions, changes in columnar (vertical) organization, and prominent dendritic growth. MRI studies of fractional anisotropy (FA) indicate microstructural changes of cortical plate (CP) during development (McKinstry et al., 2002; Takahashi et al., 2012). All these changes may contribute to the growth of CP volume during the first half of gestation when CP is recognizable as a cell dense band showing homogenous MRI signal intensity. Nevertheless, during the late mid-fetal and preterm period, when Brodmann (Brodmann, 1909) identified basic six layer lamination so called ground typus, CP shows lamination on histological sections and even on T1w MRI images and (Kostovic et al., 2008). However, the final cytoarchitectonic features are not achieved until 3 years of age (Judaš and Cepanec, 2007). Parallel with the lamination of the CP, changes occur in the organization of vertically aligned embryonic columns, which are composed of young migratory neurons (Rakic, 1988, 1995; McKinstry et al., 2002). Although our results show significant correlation between volume of the cortical plate and age, the relationship between age and the relative volume of telencephalon occupied by cortical plate is not straightforward. The cortical plate occupies the highest percentage of telencephalon in the early development (up to 20 PCW) and during the last trimester (after 30 PCW). This might be explained by the fact that from 20 to 30 PCW the subplate compartment displays a growth spurt. Furthermore, although the growth of CP until 20 PCW can be attributed to addition of neurons, it is very likely that the increase in volume of CP after 25 PCW is not caused by significant addition of new neurons (Bystron et al., 2008; Rakic, 2009; Rakic et al., 2009), although some late migratory neurons (Sanai et al., 2011) may contribute to the late developmental volume of the CP (Kostović and Judaš, 2015). Similarly, the addition of glial cells (Dobbing and Sands, 1973) is also not a massive event in the CP (Mrzljak et al., 1988, 1992). While the growth of dendrites of principal cortical neurons is accelerated after ingrowth of thalamocortical afferents (Mrzljak et al., 1988, 1992), around 24–26 PCW (Molliver et al., 1973; Kostovic and Rakic, 1990; Kostović and Judaš, 2010), the relocation of thalamocortical fibers from subplate to the CP most likely influences the shape of SP and CP volume growth curves and its thickness during late gestation.

Finally, all these factors may partly explain why we did not find significant difference in mean cortical thickness between segmented regions of cortical plate (Figure 6). This could be also due to the undetectable sub-millimeter discrete differences of immature cortex, but also due to the changes in cortical thickness that that are not detectable with our segmentation. Detailed vertex-based analysis revealed that between 16 and 21 PCW the first regions of cortical plate to become thickest are regions around central sulcus (Figure 7, upper row). Afterwards, cortical plate thickening displays central to frontal and central to occipital gradients (Figure 7, upper row). Moreover, a recent study from Huang et al. (2013) revealed that the time courses of FA drop are distinct in different brain regions during the first two trimesters of prenatal development (Huang et al., 2013). According to the authors, the FA drop during first 20 PCW is the most pronounced in the frontal cortical areas (Huang et al., 2013), which coincides with cell differentiation, cessation of neuronal migration, dendritic and axonal growth, synapse formation, and cell adhesion (Bystron et al., 2008). Thus, our results are in line with those reported previously in the literature.

### Subplate compartment

Delineation of SP from intermediate zone during mid gestation was not problematic due to the presence of the external capsule situated at the deep border of SP (Kostović and Judaš, 2002; Kostović et al., 2014), (Figures 2, 3: red dotted lines). SP is recognizable in MRI due to the hydrophilic extracellular matrix (Kostović et al., 2002; Judaš et al., 2005; Radoš et al., 2006; Widjaja et al., 2010). The delineation of the deep boundary of SP can be challenging during late gestation due to the formation of gyral white matter (Kostović et al., 2014). As well, the superficial border of SP at the interface between SP and CP is difficult to delineate during early stages due to the formation of a second CP (Kostovic and Rakic, 1990). Thus, the changing histological and histochemical properties at the interface between SP and CP, and SP and white matter (Kostovic and Rakic, 1990) certainly influence our measurements.

Despite these factors, it is evident that the volume of SP increases with age between 13 and 30 PCW, reaching the maximum around 30 PCW in most areas, occupying up to 45% of entire telencephalic volume (Figures 5, 6), and being almost 4 times thicker than CP (Figure 6). The maximal size of SP during this period may reflect an increased amount of “waiting” cortical afferents within SP, which form transient synapses before continuing into cortex (Rakic, 1977; Kostovic and Rakic, 1990; Kostović et al., 2002; Kostović and Judaš, 2007). After penetration of thalamocortical fibers into the CP, between 24–28 PCW (Kostović and Judaš, 2010), an additional convergence of associative and commissural fibers also wait in SP before entering the CP (Kostović and Judaš, 2002, 2010; Kostović and Jovanov-Milošević, 2006). Supporting evidence for this possibility is that cortical areas with absence of callosal input, such as primary visual cortex (area 17), contain thin SP while prestriate cortex shows thick SP (Kostovic and Rakic, 1984). The SP is more prominent in associative cortical areas (Figure 7, bottom row), which are strategically arranged in perisylvial cerebral territories. This supports an original hypothesis that evolutionarily, SP is related to the increased number of corticocortical connections (Kostovic and Molliver, 1974; Kostovic and Rakic, 1990; Judaš et al., 2013). Moreover, sequential ingrowth of fibers into the subplate, followed by the waiting period within the subplate, and the final relocation to the cortical plate suggests that during the peak period of fiber ingrowth the volumes of the subplate and cortical plate within same areas should be related. We therefore, expected to find a positive correlation between subplate volume and cortical plate in different anatomical regions during this developmental period (21–30 PCW) (Figure 9). The volume of the cortical plate of the frontal, occipital and parietal lobe showed positive correlation with the volume of the subplate of the same regions (Figure 9, asterisks) indicating related growth of these transient fetal compartments.

### Macroscopic development and microscopic histogenetic changes

The cerebral cortex is expanded in humans largely due to an increase number of cortical columns rather than increased cortical thickness (Rakic, 1995). However, growth of human cerebral cortex is not homogeneous in space or time. During the last trimester of human gestation, telencephalic volume, and surface area expands immensely, especially in frontal, parietal, and temporal areas (Figure 8). The vast expansion of CP occurs after the majority of neurons are born and situated in their final laminar positions, and embryonic columns are formed. This raises the question about the substrates underlying the intensive CP growth during last third of gestation we and others have observed (Retzius, 1900; His, 1904; Dobbing and Sands, 1973; O'Rahilly and Müller, 1984; Garel, 2004; Grossman et al., 2006; Trivedi et al., 2009; Habas et al., 2010a; Clouchoux et al., 2012; Lefèvre et al., 2015). During this phase, the dendrites of pyramidal neurons mature (Mrzljak et al., 1988, 1992; Marín-Padilla, 1992), cortico-cortical afferents arrive (Kostovic and Rakic, 1984; Kostović and Judaš, 2002; Kostović and Jovanov-Milošević, 2006; Kostović et al., 2014) and glia are generated (Bystron et al., 2008). The dendrites of pyramidal neurons develop rapidly after 26 PCW (Mrzljak et al., 1988, 1992) and significantly contribute to cortical volume (Petanjek et al., 2008, 2011). Development of gyral white matter (Kostović et al., 2014), which is related to the diminishment of SP (Figure 7, bottom row) and the formation of gyral convolutions (Figure 7), is also a crucial factor in the morphogenesis of late fetal cortex (Kostovic and Rakic, 1990; Kostović et al., 2014). Patterns of cortical convolutions are unique to each individual human brain (Lohmann et al., 1999). These individual patterns and the majority of gyri and sulci emerge during prenatal and early postnatal development (Connolly, 1950; Chi et al., 1977) reflecting cortical maturation as well as ingrowth of cortical afferents (Goldman and Galkin, 1978; Goldman-Rakic and Rakic, 1984). The appearance of gyri and sulci that we have observed, with the first appearance of deep fissures (sylvian, parieto-occipital, and calcarine) followed by the emergence of central sulcus (around 21 PCW), primary sulci (around 25 PCW), secondary (around 33 PCW), and tertiary sulci (around 40 PCW), is in accordance with previous descriptions from (Retzius, 1900; Connolly, 1950; Chi et al., 1977). The rapid increase of gyrification during late fetal period (Figure 7) coincides with the explosive development of corticocortical fiber connections, suggesting their possible role in gyrification (Kostovic and Rakic, 1990; Van Essen, 1997; Kostović and Jovanov-Milošević, 2006; Huang et al., 2009; Takahashi et al., 2012; Mitter et al., 2015).

## Conclusion

This study demonstrates that quantitative volumetric, surface area, and thickness data obtained by MRI-histological analysis on transient cellular compartments in the human fetal cerebrum can serve as indicators of spatio-temporal intensity of major developmental prenatal neurogenic events. The volume of proliferative compartments decrease dramatically after 25 PCW, while extracellular matrix rich synapse containing subplate compartment reached its maximum volume and thickness around 30 PCW before decreasing again. We relate this phenomenon to the pattern of growth of thalamocortical and corticocortical pathways. Moreover, during mid-gestation, the subplate zone occupied nearly half of the total hemispheric volume, indicating the relevance of the subplate compartment during human brain development. Quantitative data on cortical plate show no significant age related mean cortical thickness change, whereas surface area, volume, and level of gyrification show exponential growth during last trimester of gestation. However, as we did observe spatio-temporal areal differences in cortical thickness (vertex-wise analysis), we interpret this pattern of cortical plate differentiation as consistent with coincident differentiation of neurons, growth of dendrites, transformation of embryogenic columns, ingrowth of axons, and synaptogenesis with subsequent development of cortical convolutions. This data will improve our ability to identify transient fetal compartments in neuroimaging data of prenatal human brain.

## Limitations

There are several limitations to our study:

Firstly, the major encountered limitation is our small sample size. Secondly, as fetal brains were extracted from the skull we could not always prevent shape distortions or tissue damage that could affect some of our measurements (gyrification index and cortical plate thickness).

Thirdly, as some of the brains were available as a result of sudden infant death syndrome or especially, respiratory disease, it is possible that some damage may have occurred in these brains, potentially altering their structure.

In order to account for the known tissue shrinkage of 2.7–3.5% that is attributed to the formalin fixation (Boonstra et al., 1983; Schned et al., 1996), we reported relative volumes of transient fetal compartments. Nevertheless, we cannot rule out the minor effects of fixation on obtained absolute measures (such as the changes in cortical thickness).

We have resampled the MR images to isotropic voxel sizes of 0.15 mm (age ≤ 13 PCW) or 0.25 mm (age ≥15 PCW) because we needed to scale down from adult-size to fetal-size brains while retaining its structures (gyri and sulci). Although we corrected non-homogeneities with a small spline distance of 5 mm using the N3 method (Sled et al., 1998), we could not fully correct the non-uniformities in the images and consequently, the initial tissue classification (relying on the discrete classification using ANN (artificial neural network) and partial volume estimations) was not optimal. This led to partial volume effects that, to an extent, influenced our measures.

## Author contributions

LV Designed the study, conducted analysis, wrote the paper, and interpreted the results. CL Developed algorithm for fetal MRI image processing. MR and MP contributed to the fetal brain collection and acquisition. JG and SK contributed to data analysis and interpretation. JR and EF contributed to data analysis. MR contributed to data analysis and interpretation. PH contributed to interpretation of results. AE contributed to image processing design and interpretation of results. IK Designed the study, wrote the paper, and interpreted the results.

## Funding

The project was supported by Croatian Science Foundation (HUMANSUBPLATE) to IK and Fonds national suisse (FNS), project SPUM titled From Cortex to Classroom: Enhancing Brain Development for Premature Infants, No. 140334, to PS.

### Conflict of interest statement

The authors declare that the research was conducted in the absence of any commercial or financial relationships that could be construed as a potential conflict of interest.

## Supplementary material

Figure S1 **Semiautomatic segmentation (bottom row) of the T1 MRI images (upper row) of the 13 PCW human fetus**. Bottom row: Cortical plate in light green, intermediate zone in turquoise, proliferative compartments in pink, and subcortical gray matter with diencephalon in purple.

Figure S2 **Semiautomatic segmentation (bottom row) of the T1 MRI images (upper row) of the 20 PCW human fetus**. Bottom row: Cortical plate in light green, subplate compartment in blue, intermediate zone in turquoise, proliferative compartments in pink, and subcortical gray matter with diencephalon in purple.

Figure S3 **Semiautomatic segmentation (bottom row) of the T1 MRI images (upper row) of the 25 PCW human fetus**. Bottom row: Cortical plate in light green, subplate compartment in blue, intermediate zone in turquoise, proliferative compartments in pink, and subcortical gray matter with diencephalon in purple.
