# Supplementary figures and images for: Quantitative and Qualitative Analysis of Transient Fetal Compartments during Prenatal Human Brain Development
Source: Front Neuroanat. 2016 Feb 24;10:11. doi: 10.3389/fnana.2016.00011 (PMC4764715; doi:10.3389/fnana.2016.00011)

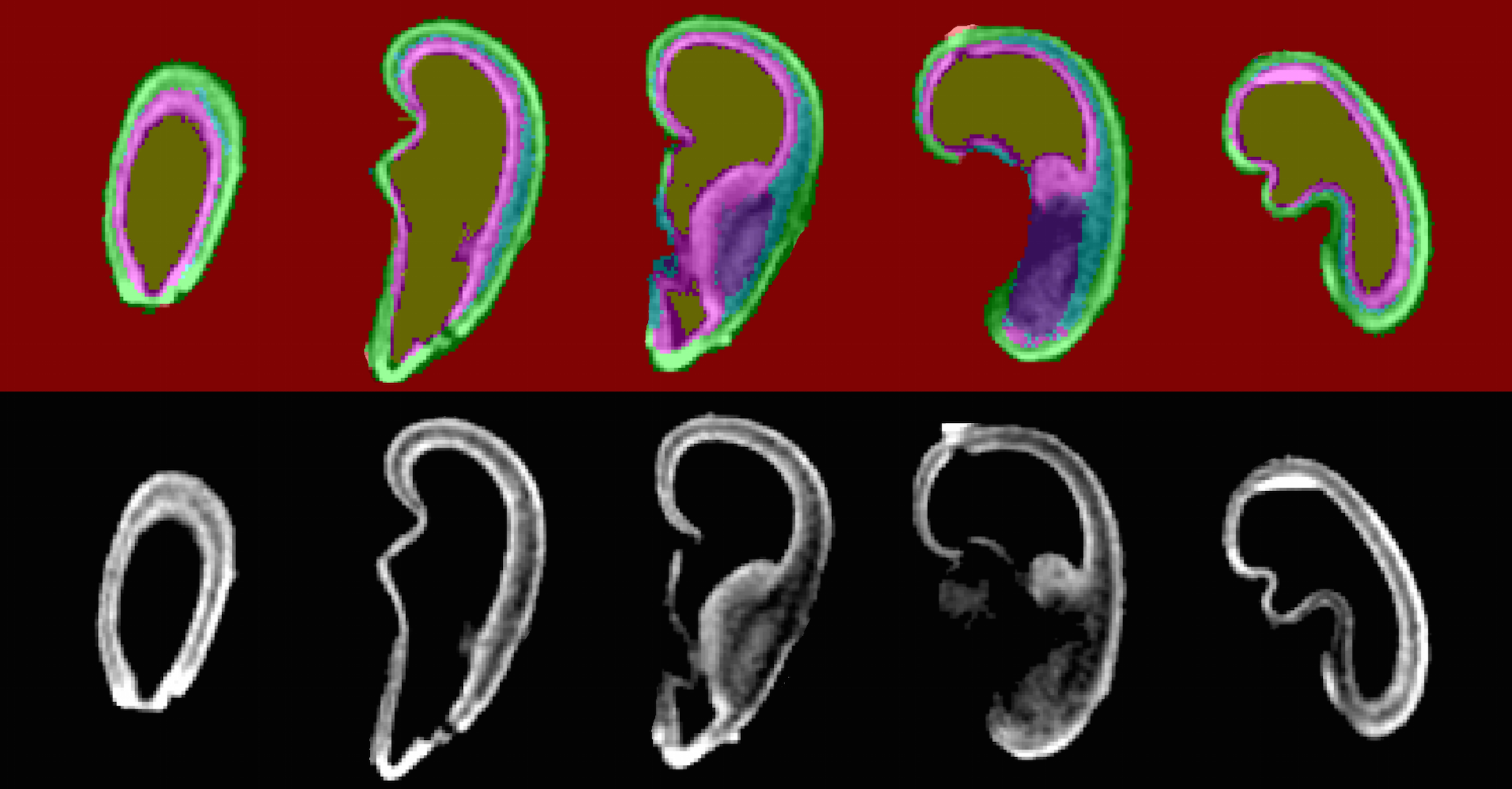

Supplement: Figure S1 — Semiautomatic segmentation (bottom row) of the T1 MRI images (upper row) of the 13 PCW human fetus. Bottom row: Cortical plate in light green, intermediate zone in turquoise, proliferative compartments in pink, and subcortical gray matter with diencephalon in purple. [file Image1.TIFF]

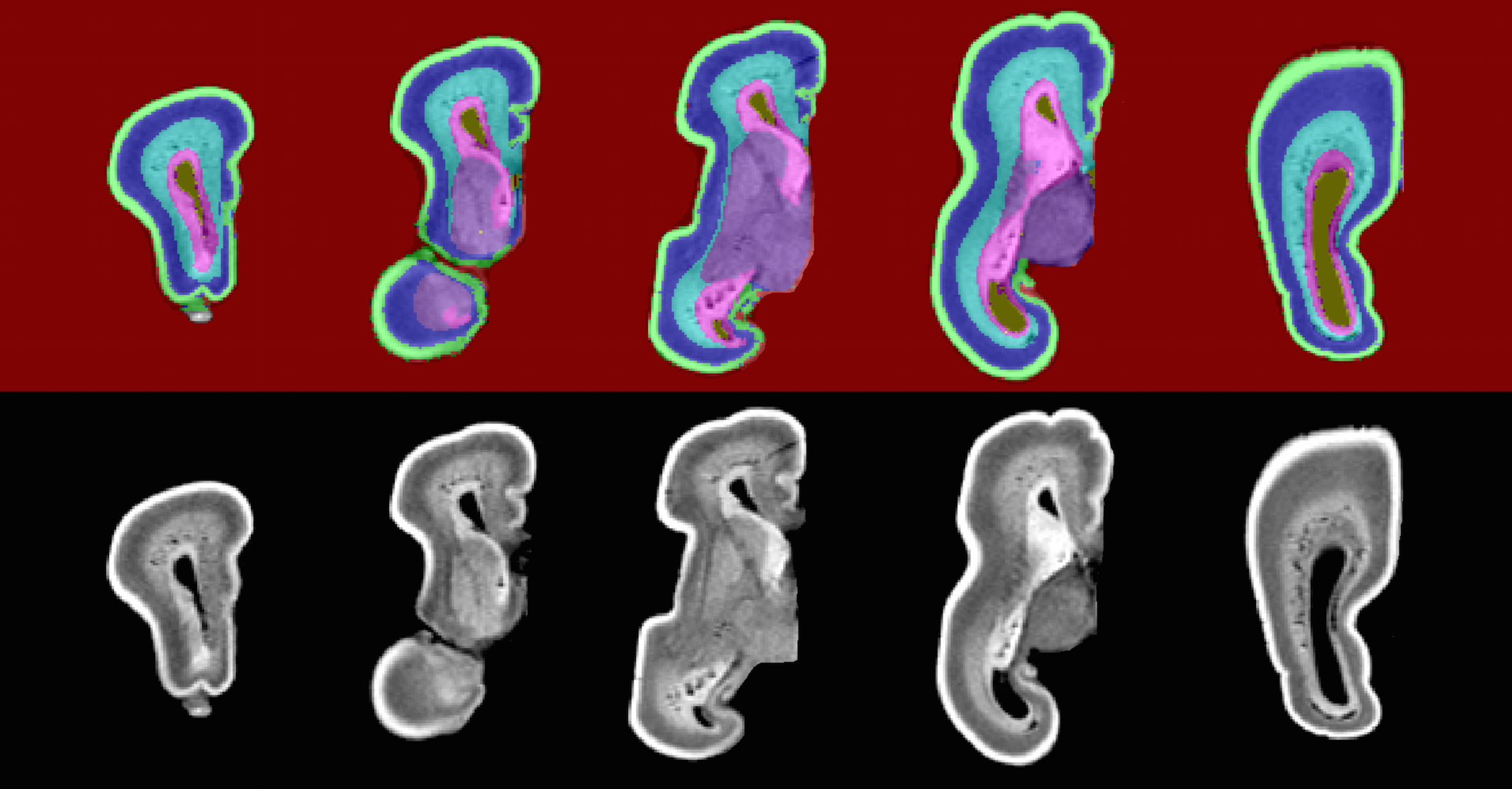

Supplement: Figure S2 — Semiautomatic segmentation (bottom row) of the T1 MRI images (upper row) of the 20 PCW human fetus. Bottom row: Cortical plate in light green, subplate compartment in blue, intermediate zone in turquoise, proliferative compartments in pink, and subcortical gray matter with diencephalon in purple. [file Image2.TIFF]

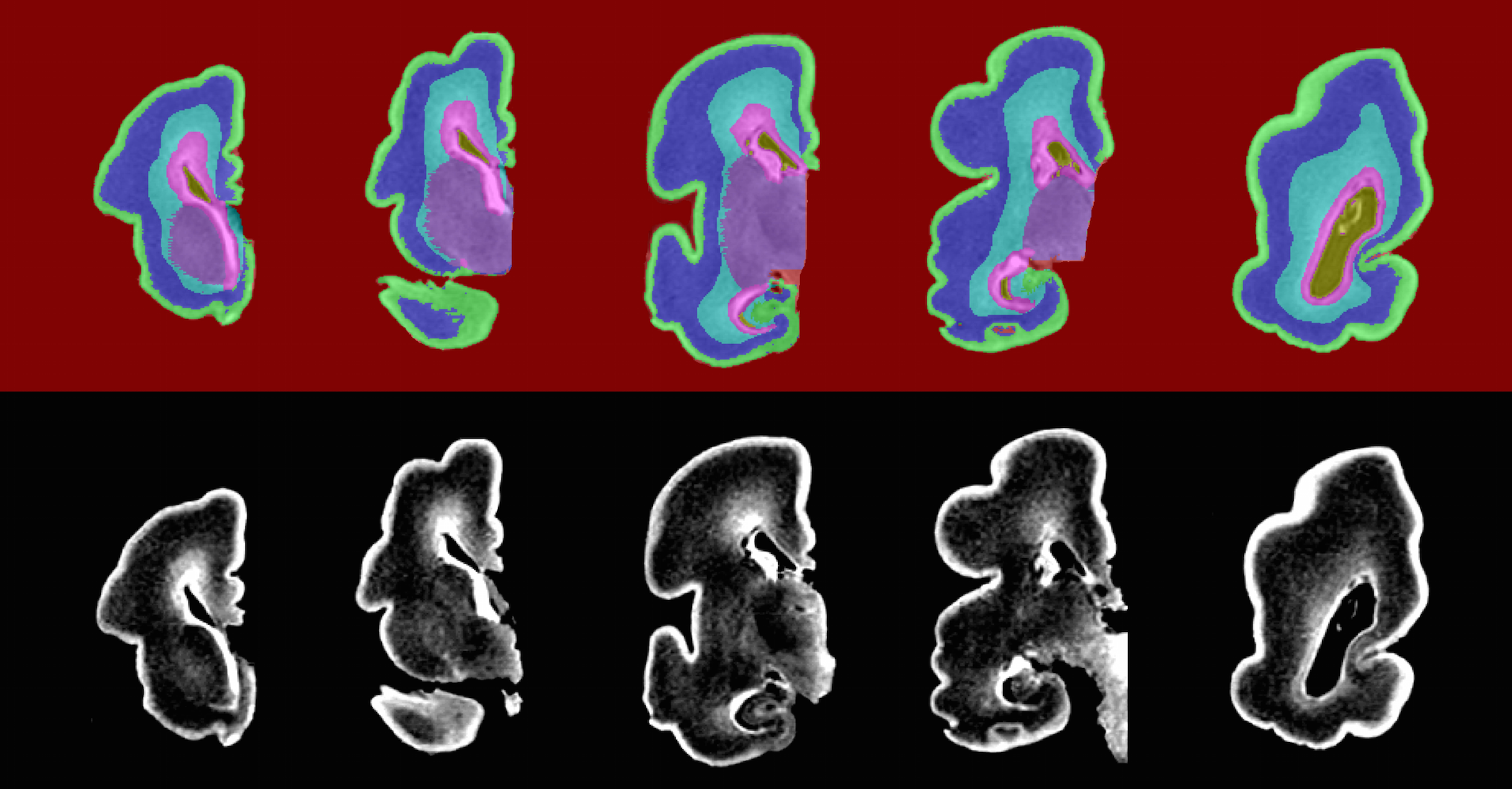

Supplement: Figure S3 — Semiautomatic segmentation (bottom row) of the T1 MRI images (upper row) of the 25 PCW human fetus. Bottom row: Cortical plate in light green, subplate compartment in blue, intermediate zone in turquoise, proliferative compartments in pink, and subcortical gray matter with diencephalon in purple. [file Image3.TIFF]
